# Correlations between inflammatory biomarkers and clinicopathological features in surgically resected thymic epithelial tumors: implications for preoperative diagnosis

**DOI:** 10.7717/peerj.21232

**Published:** 2026-05-11

**Authors:** Jun Fu, Peng Han, Jiahang Xu, Wanjun Deng, Baojun Chen, Yong Liu

**Affiliations:** 1 Thoracic Surgery, The Central Hospital of Wuhan, Tongji Medical College, Huazhong University of Science and Technology, Wuhan, Hubei, China; 2 Tongji Medical College, Huazhong University of Science and Technology, Key Laboratory for Molecular Diagnosis of Hubei Province, The Central Hospital of Wuhan, Wuhan, Hubei, China; 3 Fudan University Shanghai Cancer Center, Shanghai, China

**Keywords:** Thymic epithelial tumors, NLR, PLR, SII

## Abstract

**Objective:**

This study aimed to analyze the correlations between pathological characteristics, risk factors, and inflammatory biomarkers in patients with thymic epithelial tumors (TETs) undergoing surgical resection.

**Methods:**

A retrospective cohort of adult patients with histopathologically confirmed TETs undergoing radical resection between 2015 and 2024 was analyzed. The Kruskal–Wallis test was employed to evaluate associations of systemic immune-inflammation index (SII), platelet-to-lymphocyte ratio (PLR), and neutrophil-to-lymphocyte ratio (NLR) with clinical features (age; gender; myasthenia gravis (MG); World Health Organization (WHO) stage; tumor size; Masaoka stage; WHOMScore). A nomogram diagnostic model was constructed to differentiate thymoma from thymic carcinoma.

**Results:**

A total of 111 patients (52 males, 59 females) were enrolled, including eight cases of type A thymoma, 38 type AB, nine type B1, 16 type B2, 12 type B3, and 28 thymic carcinomas. Correlations between SII, NLR, PLR, and clinicopathological features (gender, age, MG, tumor size, risk stratification, Masaoka stage, and WHO classification) were analyzed. Significant differences in SII, NLR, and PLR were observed across WHO classifications (*P*_SII_ < 0.001; *P*_NLR_ < 0.001; *P*_PLR_ = 0.004). Dunn’s test further revealed that these differences primarily stemmed from distinctions between thymoma and thymic carcinoma groups. Based on PLR and MG status, a combined diagnostic model was developed, which demonstrated favorable performance in differentiating thymoma from thymic carcinoma (AUC = 0.804).

**Conclusion:**

Our findings suggest that inflammatory biomarkers (SII, NLR, and PLR) are significantly associated with the malignancy of TETs. The combination of PLR and MG status may serve as a useful auxiliary tool for distinguishing thymoma from thymic carcinoma, showing potential for clinical application.

## Introduction

The thymus, primarily composed of epithelial cells and lymphocytes, serves as the anatomical site where precursor cells migrate and differentiate into lymphocytes (Elm & Levidou, 2024). Thymic epithelial tumors (TETs) represent a relatively rare neoplastic family and constitute the most prevalent malignancies arising from thymic epithelial tissues, accounting for approximately 0.2%–1.5% of all human cancers (Conforti et al., 2020). The predominant thymic neoplasms are broadly categorized into two groups: thymomas and thymic carcinomas (TCs) (Damaj et al., 2025; Falkson et al., 2022; Tateo et al., 2020). Histoimmunologically, TETs demonstrate variable proportions of neoplastic thymic epithelial cells intermixed with non-neoplastic lymphocytes, with distinct cytological characteristics and biological behaviors observed between thymomas and TCs (Prays & Ortiz-Villalón, 2022).

The latest World Health Organization (WHO) classification system stratifies TETs into six subtypes (A, AB, B1, B2, B3, and C) based on the degree of malignant differentiation in epithelial cells and lymphocyte proportion. The first four subtypes (A, AB, B1, B2) constitute thymoma variants characterized by relatively well-differentiated histology and lower biological aggressiveness (Marx et al., 2022; Roden et al., 2022). Type C primarily denotes TCs, which exhibit marked invasiveness and metastatic potential. Histogenetically, type A tumors originate from medullary phenotype epithelium, while all B subtypes derive from cortical epithelium. The B3 subtype, termed well-differentiated TC, represents a clinicopathological entity with intermediate features between thymomas and type C carcinomas (Kuhn et al., 2023; Lee et al., 2016). Furthermore, the 2015 WHO classification system also categorizes thymomas into two major groups: low-risk (types A, AB, and B1) (LRT) and high-risk (types B2 and B3) (HRT). Complementing the WHO classification, the Masaoka-Koga staging system remains the most widely accepted prognostic framework, defining TETs as four stages (I, II, III, and IV stage). Both WHO histological classification and Masaoka-Koga staging demonstrate significant correlations with survival outcomes (Kuhn et al., 2023; Lee et al., 2016; Mendogni et al., 2022; Sato et al., 2018).

Surgical resection remains the cornerstone of TET management, where complete tumor excision with involved organs constitutes the therapeutic objective and significantly influences postoperative recurrence-free survival (Falkson et al., 2022; Falkson et al., 2023; Huang et al., 2021). Preoperative imaging modalities such as contrast-enhanced computed tomography (CT) and magnetic resonance imaging (MRI) demonstrate reasonable accuracy in distinguishing thymomas from benign thymic cysts, yet exhibit limitations in differentiating thymomas from TCs (Dai et al., 2023; Li et al., 2023; Liu et al., 2021). Intraoperative visual assessment of tumor invasiveness remains subjective, underscoring the clinical need for precise preoperative staging biomarkers. The establishment of objective evaluation criteria could optimize surgical planning and potentially reduce recurrence rates.

Emerging evidence highlights significant associations between tumor-associated immune responses and oncological progression (Ao et al., 2023; Masaoutis et al., 2022; Tateo et al., 2020). Cytokines produced by malignant cells or tumor microenvironments stimulate systemic inflammation, manifesting as elevated circulating peripheral blood cells including neutrophils, lymphocytes, monocytes, and platelets (Huang et al., 2022; Okada et al., 2020; Veraar et al., 2019). Numerous studies have validated the prognostic value of inflammatory indices such as neutrophil-to-lymphocyte ratio (NLR), systemic immune-inflammation index (SII), and platelet-to-lymphocyte ratio (PLR) across various malignancies including gastric carcinoma (Çağlar, 2023; Wu et al., 2025), colorectal cancer (Yamamoto, Kawada & Obama, 2021; Yang et al., 2023), lung cancer (Mandaliya et al., 2019), bladder cancer (Salari et al., 2024), and breast cancer (Morkavuk, Kocaöz & Korukluoğlu, 2021; Savioli et al., 2022).

To date, no investigations have elucidated the relationship between these inflammatory biomarkers and TET staging. This retrospective study therefore aims to evaluate the correlation of NLR, SII, and PLR with TET staging parameters, with the objective of developing preoperative predictive tools to guide surgical decision-making through evidence-based stratification.

### Method

### Study population

This retrospective investigation received ethical approval from the Ethics Committee of Wuhan Central Hospital (Approval No. WHZXKYL2025-055), and the requirement for informed consent was waived. We systematically analyzed pathological records of consecutive patients who underwent complete surgical resection for thymic epithelial tumors at our institution between January 2015 and December 2024. The study cohort was selected according to predefined criteria: (i) histologically confirmed diagnosis of thymoma or TC through postoperative pathological examination; (ii) availability of comprehensive preoperative serological profiles. Exclusion criteria encompassed: (i) recent steroid therapy within 4 weeks preceding surgery; (ii) concurrent active infections, autoimmune disorders, or hematological malignancies; (iii) history of blood transfusion; (iv) documented history of other malignancies; (v) incomplete clinicopathological documentation, laboratory parameters, or follow-up records.

### Inflammatory biomarkers and diagnostic model

Data were retrospectively extracted from institutional medical records. All serological data were obtained from the lab tests closest to the time of surgery within one week prior to the procedure. The NLR was calculated as the absolute neutrophil count divided by the absolute lymphocyte count in peripheral blood. The SII was computed as platelet count × neutrophil count/lymphocyte count. The PLR was derived from platelet count divided by lymphocyte count. Pearson correlation coefficient exceeding 0.9 between variables was considered indicative of high multicollinearity. The WHOMscore was operationalized according to the methodology described by Lee et al. (2016) (Table S1). This scoring system constitutes a risk stratification model for postoperative recurrence of TETs, integrating both Masaoka-Koga staging and WHO histological classification. A higher score indicates an elevated risk of recurrence. Variable selection was performed to identify the most parsimonious set of predictors using a backward elimination approach based on the Akaike Information Criterion (AIC). This procedure was implemented using the step() function from the rms package in R. Receiver-operating characteristic (ROC) curve analysis was employed to evaluate the diagnostic discrimination of these biomarkers between thymomas and TCs. A predictive nomogram was subsequently constructed by integrating significant biomarkers into a multivariate logistic regression model, enabling visual quantification of malignancy risk stratification. Model calibration, which denotes the agreement between predicted probabilities and observed outcomes, was evaluated by plotting calibration curves and performing the Hosmer–Lemeshow goodness-of-fit test. Bootstrap resampling with 1,000 resamples was performed for internal validation.

### Statistical analysis

All statistical analyses were performed using R software (version 4.0.1). Continuous variables were first assessed for normality using the Shapiro–Wilk test. Non-normally distributed variables are presented as median (interquartile range, IQR), while normally distributed variables are presented as mean ± standard deviation (SD). Platelet values were normally distributed (Shapiro–Wilk test, *P* > 0.05), and comparisons between groups were performed using the *t*-test (for binary variables) or one-way analysis of variance (ANOVA) (for categorical variables with more than two groups). Inflammatory markers (SII, NLR, PLR), neutrophils, and lymphocytes exhibited skewed distributions (Shapiro–Wilk test, *P* < 0.05), and differences among groups were compared using the Kruskal–Wallis test. Dunn’s *post-hoc* test was used for pairwise comparisons between inflammatory markers and WHO stages, with *P*-values adjusted using the Bonferroni method for multiple comparisons. The statistical methods for diagnostic model development and validation were consistent with those described above. An adjusted *P*-value (Padj) < 0.05 was considered statistically significant for multiple comparisons. All statistical tests were two-tailed, and a *P*-value < 0.05 was considered statistically significant. The following R packages were used for statistical analyses: FSA, tidyverse, xtable, rms, pROC, tableone, and gtsummary.

## Results

### Patient characteristics

This retrospective study included 111 surgically resected TETs with clinicopathological characteristics detailed in Table 1. The cohort comprised 52 males and 59 females (median age 56 years, range 26–78), including 22 thymoma patients with paraneoplastic MG. Tumor size distribution showed 100 cases (90.1%) with tumors <three cm and 11 cases (9.9%) with tumors ≥ three cm. Histological subtypes per WHO classification were: Type A (*n* = 8), AB (*n* = 38), B1 (*n* = 9), B2 (*n* = 16), B3 (*n* = 12), and TC (*n* = 28). Stratification identified low-risk thymoma (*n* = 54), high-risk thymoma (*n* = 30), and TC (*n* = 27). Masaoka-Koga staging distributed as I (*n* = 58), II (*n* = 32), III (*n* = 19), and IV (*n* = 2), with advanced stages (III/IV) combined for analysis. The WHOMScore system categorized cases into Stage I (*n* = 52), II (*n* = 36), and III (*n* = 23).

**Table 1:** Clinical and pathological findings in thymic epithelial tumor cases. This presents the distribution of clinical and pathological characteristics among 111 patients with surgically resected thymic epithelial tumors (TETs).

Variable	Overall *N* = 111
Sex (%)	
Female	59 (53.2%)
Male	52 (46.8%)
Age (median (range))	56 (26-78)
Myasthenia gravis (%)	
No	89 (80.2%)
Yes	22 (19.8%)
WHO stage (%)	
A	8 (7.2%)
AB	38 (34.2%)
B1	9 (8.1%)
B2	16 (14.4%)
B3	12 (10.8%)
C	28 (25.2%)
Risk degree (%)	
LRT	54 (48.6%)
HRT	30 (27.0%)
TC	27 (24.3%)
Tumor size (%)	
<3 cm	100 (90.1%)
≥3 cm	11 (9.9%)
Masaoka stage (%)	
I	58 (52.3%)
II	32 (28.8%)
III-IV	21 (18.9%)
WHOMScore (%)	
I	52 (46.8%)
II	36 (32.4%)
III	23 (20.7%)

**Notes.**

LRT, low-risk (types A, AB, and B1); HRT, high-risk (types B2 and B3); TC, thymic carcinomas.

**Table 2:** Statistical analysis of inflammatory markers and clinical parameters. This depicts the distribution of platelets, neutrophils, and lymphocytes across different clinical and pathological characteristics.

Variable	Overall *N* = 111	Platelets		Neutrophils		Lymphocytes	
		Mean (SD)	*P*	Median (IQR)	*P*	Median (IQR)	*P*
Sex (%)							
female	59 (53.2%)	222.29 (68.61)	0.098	2.99 (2.43, 3.81)	**0.001**	1.69 (1.36, 2.21)	0.451
male	52 (46.8%)	242.52 (58.90)		3.99 (3.21, 5.23)		1.89 (1.37, 2.42)	
Age (mean (SD))							
<60	63(56.8%)	236.41 (70.08)	0.376	3.53 (2.72, 4.37)	0.953	1.84 (1.36, 2.42)	0.232
≥60	48(43.2%)	225.67 (57.18)		3.39 (2.83, 4.46)		1.69 (1.36, 2.04)	
Myasthenia gravis (%)							
No	89 (80.2%)	227.72 (67.15)	0.13	3.49 (2.84, 4.18)	0.559	1.73 (1.36, 2.28)	0.827
Yes	22 (19.8%)	248.14 (52.17)		3.16 (2.71, 5.47)		1.63 (1.46, 2.34)	
WHO stage (%)							
A	8 (7.2%)	244.38 (55.77)	**0.011**	3.24 (2.25, 5.19)	0.408	1.70 (1.12, 2.20)	0.076
AB	38 (34.2%)	228.16 (66.72)		3.67 (2.96, 4.74)		1.81 (1.46, 2.29)	
B1	9 (8.1%)	192.22 (75.84)		2.91 (1.99, 3.39)		2.15 (1.79, 2.47)	
B2	16 (14.4%)	245.38 (60.90)		3.21 (2.54, 4.65)		2.00 (1.59, 2.41)	
B3	12 (10.8%)	187.50 (38.13)		3.27 (2.78, 4.51)		1.89 (1.23, 2.52)	
C	28 (25.2%)	256.96 (59.97)		3.81 (2.95, 4.34)		1.49 (1.25, 1.84)	
Risk degree (%)							
LRT	54 (48.6%)	221.67 (57.58)	0.066	3.21 (2.67, 4.29)	0.643	2.05 (1.37, 2.38)	**0.012**
HRT	30 (27.0%)	224.83 (68.05)		3.51 (2.81, 4.37)		1.84 (1.44, 2.31)	
TC	27 (24.3%)	256.85 (61.11)		3.78 (2.90, 4.50)		1.48 (1.24, 1.81)	
Tumor size (%)							
<3 cm	100 (90.1%)	228.59 (67.04)	0.886	3.42 (2.66, 4.50)	0.487	1.81 (1.44, 2.31)	0.756
≥3 cm	11 (9.9%)	246.20 (52.18)		3.75 (2.91, 4.18)		1.41 (1.11, 1.88)	
Masaoka stage (%)							
I	58 (52.3%)	223.84 (69.61)	0.39	3.24 (2.67, 4.13)	0.417	1.69 (1.42, 2.26)	0.198
II	32 (28.8%)	242.53 (58.60)		3.69 (2.74, 5.08)		1.99 (1.47, 2.31)	
III-IV	21 (18.9%)	237.24 (59.43)		3.65 (2.91, 4.15)		1.57 (1.21, 1.96)	
WHOMScore (%)							
I	52 (46.8%)	223.19 (66.41)	0.425	3.51 (2.73, 4.53)	0.94	1.84 (1.43, 2.32)	0.084
II	36 (32.4%)	238.47 (64.72)		3.37 (2.76, 4.25)		1.75 (1.49, 2.28)	
III	23 (20.7%)	240.65 (61.24)		3.44 (2.87, 4.50)		1.37 (1.13, 2.04)	

**Notes.**

LRT, low-risk (types A, AB, and B1); HRT, high-risk (types B2 and B3); TC, thymic carcinomas. Normal reference ranges: Platelets, (100–300) × 10^9^/L; Neutrophils, 1.8–6.3 × 10^9^/L; Lymphocytes, 0.8–4.0 × 10^9^/L. Bold values indicate significant differences between groups (*p* < 0.05).

### Distribution profiles of platelets, neutrophils, and lymphocytes in thymoma patients

Statistical analysis of inflammatory markers and clinical parameters (Table 2) revealed significantly lower neutrophil counts in females compared to males (*P* < 0.01), while platelet and lymphocyte levels showed no sex-based differences. Age and MG comorbidity status demonstrated no significant associations with platelet, neutrophil, or lymphocyte counts. Further stratification analysis identified differential expression patterns across pathological classifications: platelet levels varied significantly by WHO histological classification (*P* = 0.011), whereas lymphocyte counts exhibited associations with risk stratification (*P* = 0.012) and WHOMScore stages (*P* = 0.084). No significant correlations were observed between these hematological parameters and Masaoka staging or tumor size.

### Distribution profiles of SII, NLR, and PLR in thymoma patients

We systematically quantified SII, NLR, and PLR across the cohort, with detailed variations across clinicopathological parameters presented in Table 3. Analysis revealed significantly elevated SII levels in male patients *versus* females (*P* < 0.05), whereas NLR and PLR showed no sex-based variations. These inflammatory indices demonstrated no significant associations with age or MG comorbidity. Notably, all three biomarkers exhibited differential expression patterns across WHO histological classifications (*P*_SII_ < 0.001; *P*_NLR_ < 0.001; *P*_PLR_ = 0.004) and risk stratification categories (*P*_SII_ = 0.001; *P*_NLR_ = 0.005; *P*_PLR_ < 0.001). No correlations were observed with Masaoka staging and tumor size, while WHOMScore analysis identified PLR as the sole parameter demonstrating statistical significance (*P* = 0.015).

**Table 3:** The variations of SII, NLR and PLR in thymic epithelial tumor cases. This describes the distribution of SII, NLR, and PLR across different clinical and pathological characteristics.

Variable	Overall N=111	SII		NLR		PLR	
		Median (IQR)	*P*	Median (IQR)	*P*	Median (IQR)	*P*
Sex (%)							
female	59 (53.2%)	375.86 (275.02, 650.83)	**0.01**	1.92 (1.47, 2.31)	0.063	121.23 (96.82, 151.40)	0.73
male	52 (46.8%)	511.45 (387.63, 757.68)		2.19 (1.62, 2.73)		130.94 (99.12, 176.88)	
Age (mean (SD))							
<60	63(56.8%)	416.34 (278.98, 670.17)	0.934	1.86 (1.48, 2.49)	0.476	119.70 (94.81, 161.15)	0.76
≥60	48(43.2%)	421.27 (358.62, 654.66)		2.21 (1.80, 2.57)		132.98 (102.78, 169.83)	
Myasthenia gravis (%)							
No	89 (80.2%)	410.97 (316.80, 650.83)	0.159	1.98 (1.50, 2.51)	0.284	121.23 (96.82, 161.15)	0.26
Yes	22 (19.8%)	475.53 (359.49, 791.53)		2.04 (1.63, 3.09)		142.50 (103.98, 171.23)	
WHO stage (%)							
A	8 (7.2%)	431.39 (319.95, 825.03)	**<0.001**	1.72 (1.33, 3.46)	**<0.001**	146.30 (119.02, 163.20)	**0.004**
AB	38 (34.2%)	411.99 (340.98, 634.42)		2.10 (1.55, 2.47)		115.27 (92.45, 146.40)	
B1	9 (8.1%)	244.57 (195.91, 357.75)		1.28 (1.00, 1.89)		89.92 (77.10, 98.45)	
B2	16 (14.4%)	409.02 (301.39, 767.95)		1.75 (1.42, 2.80)		126.86 (99.02, 152.90)	
B3	12 (10.8%)	373.15 (275.44, 410.57)		1.84 (1.45, 2.18)		103.25 (76.95, 138.46)	
C	28 (25.2%)	654.07 (444.33, 796.02)		2.47 (1.97, 2.71)		172.82 (135.54, 219.98)	
Risk degree (%)							
LRT	54 (48.6%)	379.09 (285.97, 543.50)	**<0.001**	1.72 (1.35, 2.29)	**0.005**	110.62 (90.17, 149.17)	**<0.001**
HRT	30 (27.0%)	409.31 (275.02, 605.85)		1.91 (1.47, 2.40)		115.27 (91.98, 146.40)	
TC	27 (24.3%)	657.30 (445.06, 800.50)		2.50 (1.98, 2.83)		174.40 (138.22, 222.14)	
Tumor size (%)							
<3 cm	100 (90.1%)	410.97 (296.84, 650.83)	0.726	1.95 (1.48, 2.50)	0.462	119.70 (94.81, 149.38)	0.949
≥3 cm	11 (9.9%)	611.17 (410.57, 804.01)		2.45 (1.84, 3.62)		178.47 (108.64, 222.41)	
Masaoka stage (%)							
I	58 (52.3%)	409.31 (328.21, 584.42)	0.12	1.98 (1.54, 2.51)	0.247	116.14 (92.45, 151.40)	0.136
II	32 (28.8%)	447.57 (291.41, 797.72)		1.95 (1.48, 2.38)		127.82 (101.82, 150.09)	
III-IV	21 (18.9%)	527.43 (392.94, 791.53)		2.43 (1.71, 3.41)		158.39 (103.23, 217.82)	
WHOMScore (%)							
I	52 (46.8%)	409.31 (273.00, 612.61)	0.114	1.91 (1.45, 2.39)	0.279	115.27 (91.29, 144.10)	**0.015**
II	36 (32.4%)	426.63 (343.85, 682.64)		2.04 (1.56, 2.55)		125.29 (99.12, 162.42)	
III	23 (20.7%)	583.29 (371.82, 816.48)		2.24 (1.71, 3.41)		158.39 (114.04, 222.14)	

**Notes.**

LRT, low-risk (types A, AB, and B1); HRT, high-risk (types B2 and B3); TC, thymic carcinomas. NLR, neutrophil-to-lymphocyte ratio; PLR, platelet-to-lymphocyte ratio; SII, systemic immune-inflammation index. Bold values indicate significant differences between groups (*p* < 0.05).

### Distribution profiles of SII, NLR, and PLR in WHO histological classifications

**Figure 1:**
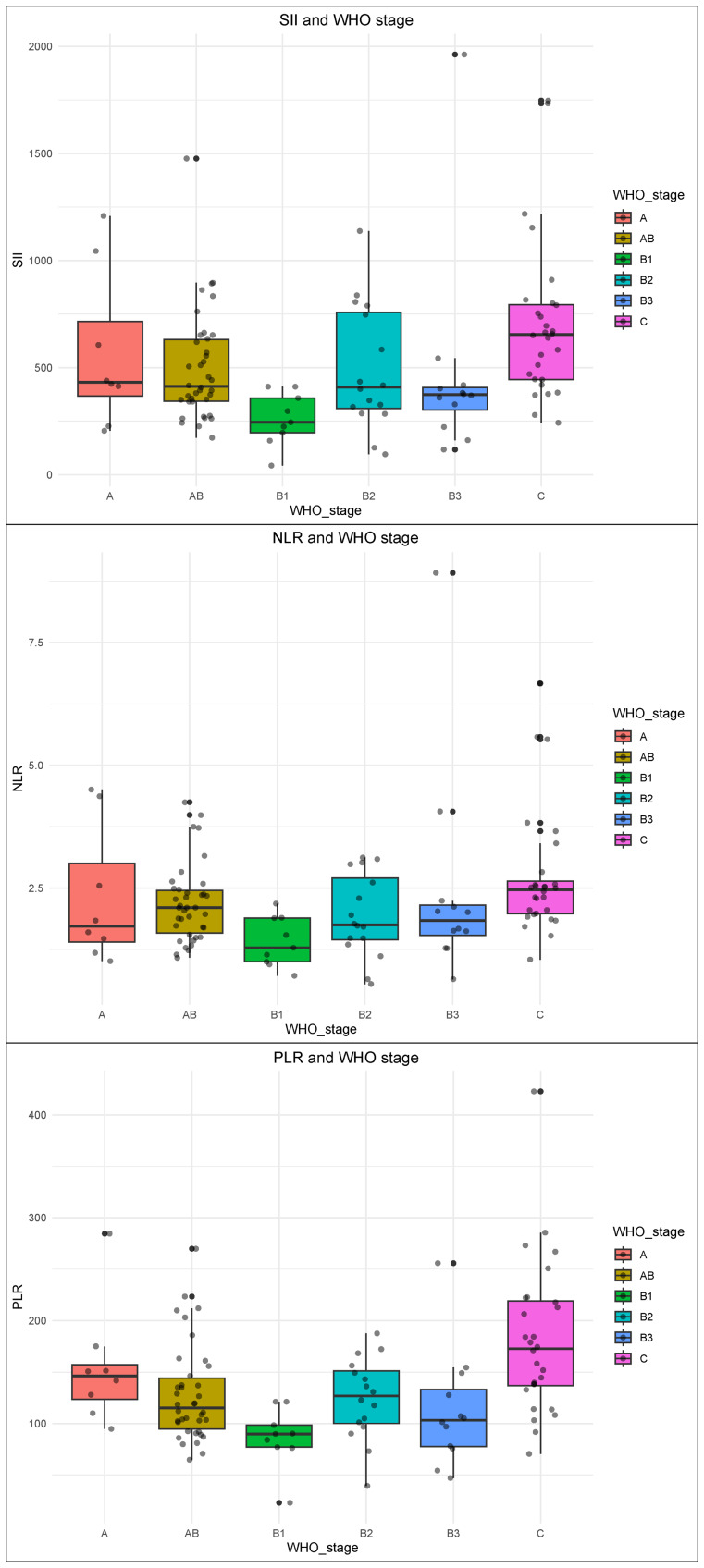
Distribution profiles of SII, NLR, and PLR in WHO histological classifications. Distribution of SII, NLR, and PLR across thymoma types A, AB, B1, B2, B3, and thymic carcinoma (TC), with PLR showing significantly higher expression in TC (*P* < 0.05).

**Figure 2:**
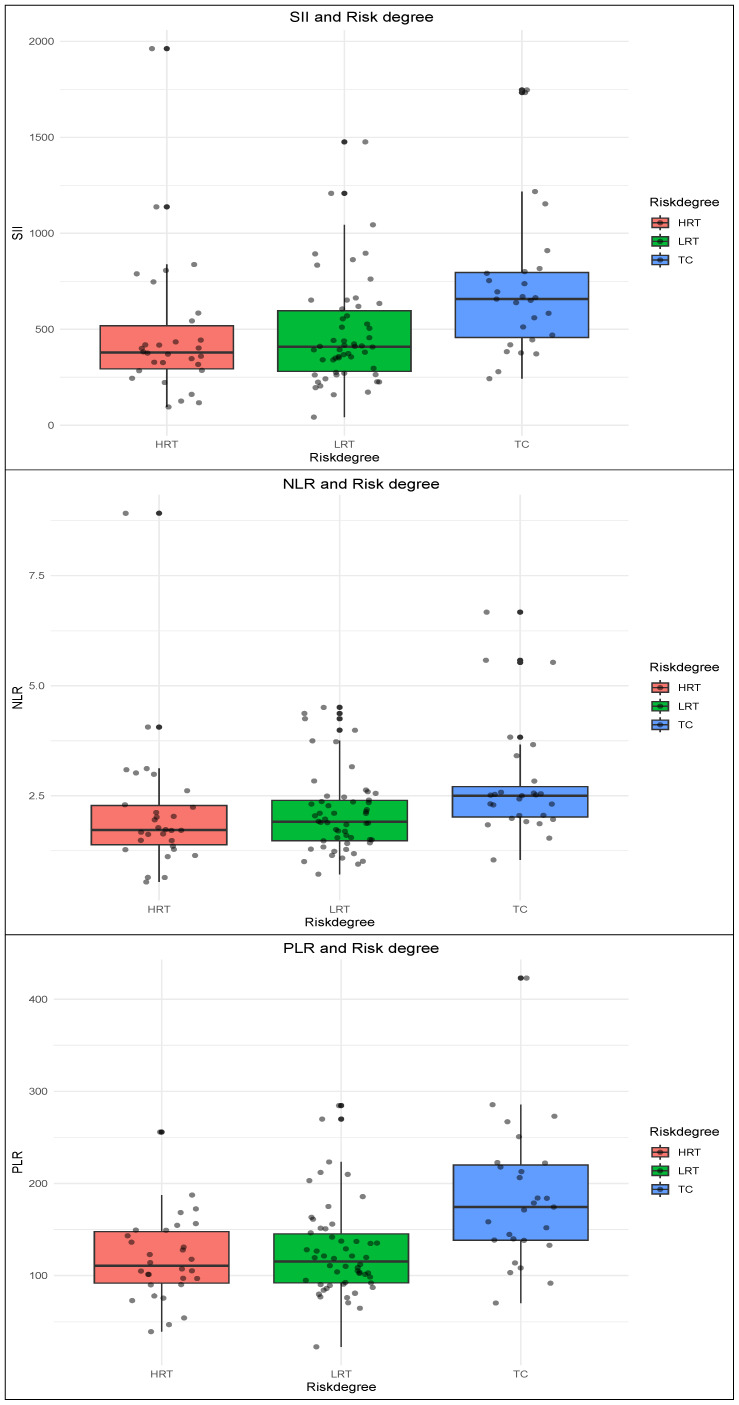
Distribution profiles of SII, NLR, and PLR in risk stratification. Distribution of SII, NLR, and PLR across high-risk thymoma (HRT), low-risk thymoma (LRT), and thymic carcinoma (TC). All three biomarkers were significantly elevated in TC (*P* < 0.05).

Dunn’s test was conducted to further investigate variations in immune-inflammatory biomarkers across subgroups. The results demonstrated significant elevations in SII, PLR, and NLR within TC compared to other WHO histological subtypes, with PLR exhibiting the most prominent differential expression (Fig. 1). Specifically, SII levels in TC were significantly higher *versus* B1 (*P* < 0.001) and B3 subtypes (*P* = 0.027), while PLR showed marked increases in TC relative to AB (*P* = 0.012), B1 (*P* < 0.001), and B3 subtypes (*P* = 0.01). NLR displayed TC-specific elevations compared to B1 subtype (*P* < 0.001) (Table 4). Consistent with these findings, risk stratification analysis revealed that all three biomarkers were significantly elevated in TC compared to both high-risk thymoma (HRT) and low-risk thymoma (LRT) (*P* < 0.05), whereas no inter-group differences were observed between HRT and LRT (Fig. 2, Table 5).

Based on these distinct inflammatory profiles, patients were stratified into thymoma (*n* = 83) and TC (*n* = 28) cohorts. Comparative analysis confirmed that TC exhibited significantly elevated levels of SII (*P* = 0.006), NLR (*P* = 0.025), and PLR (*P* < 0.001), highlighting the unique systemic inflammatory signature associated with TC (Table 6).

### Construction of clinical diagnostic models

**Figure 3:**
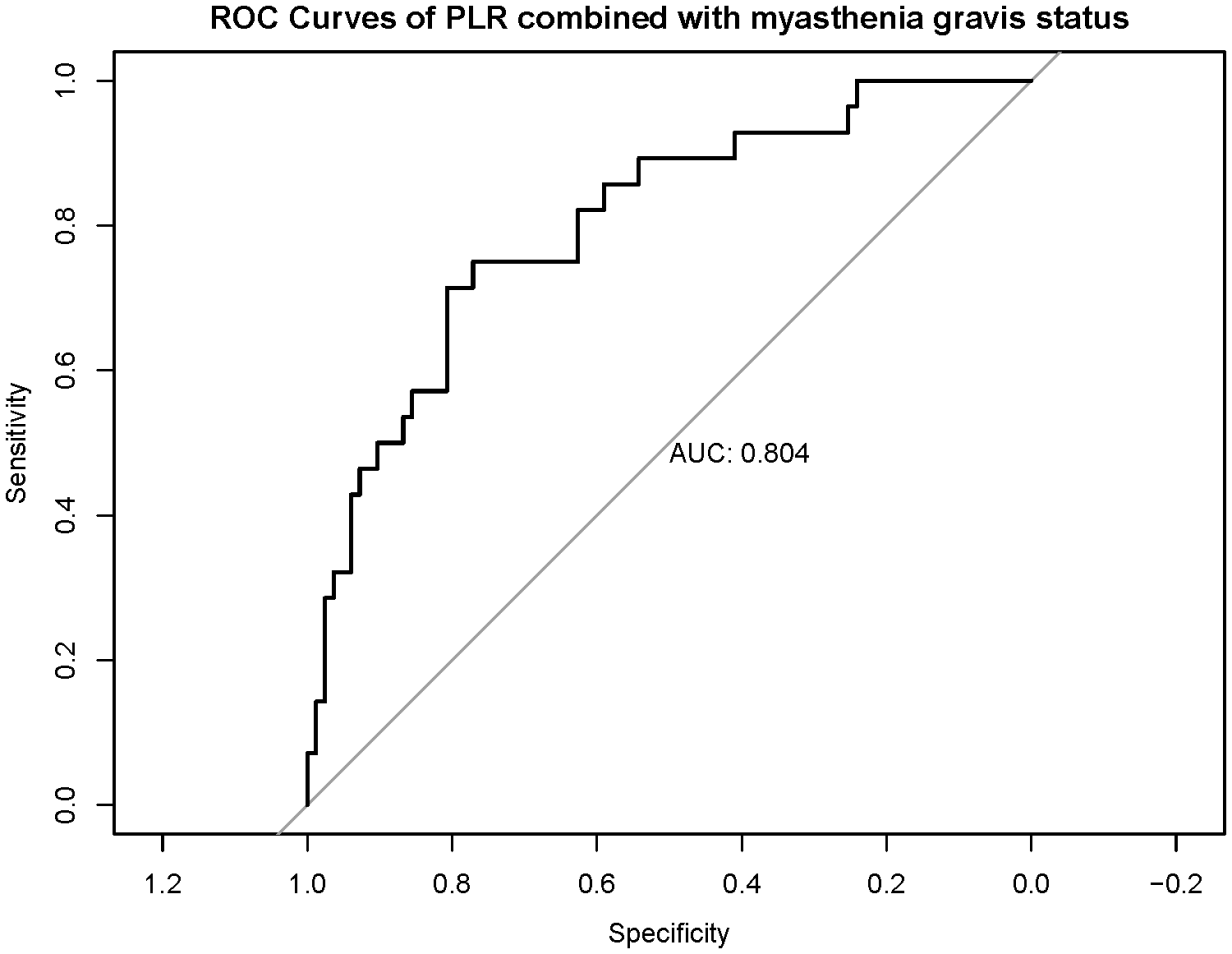
The receiver operating characteristic curve analysis. ROC curve analysis demonstrated that the PLR + MG model exhibited favorable diagnostic performance (AUC = 0.804).

**Figure 4:**
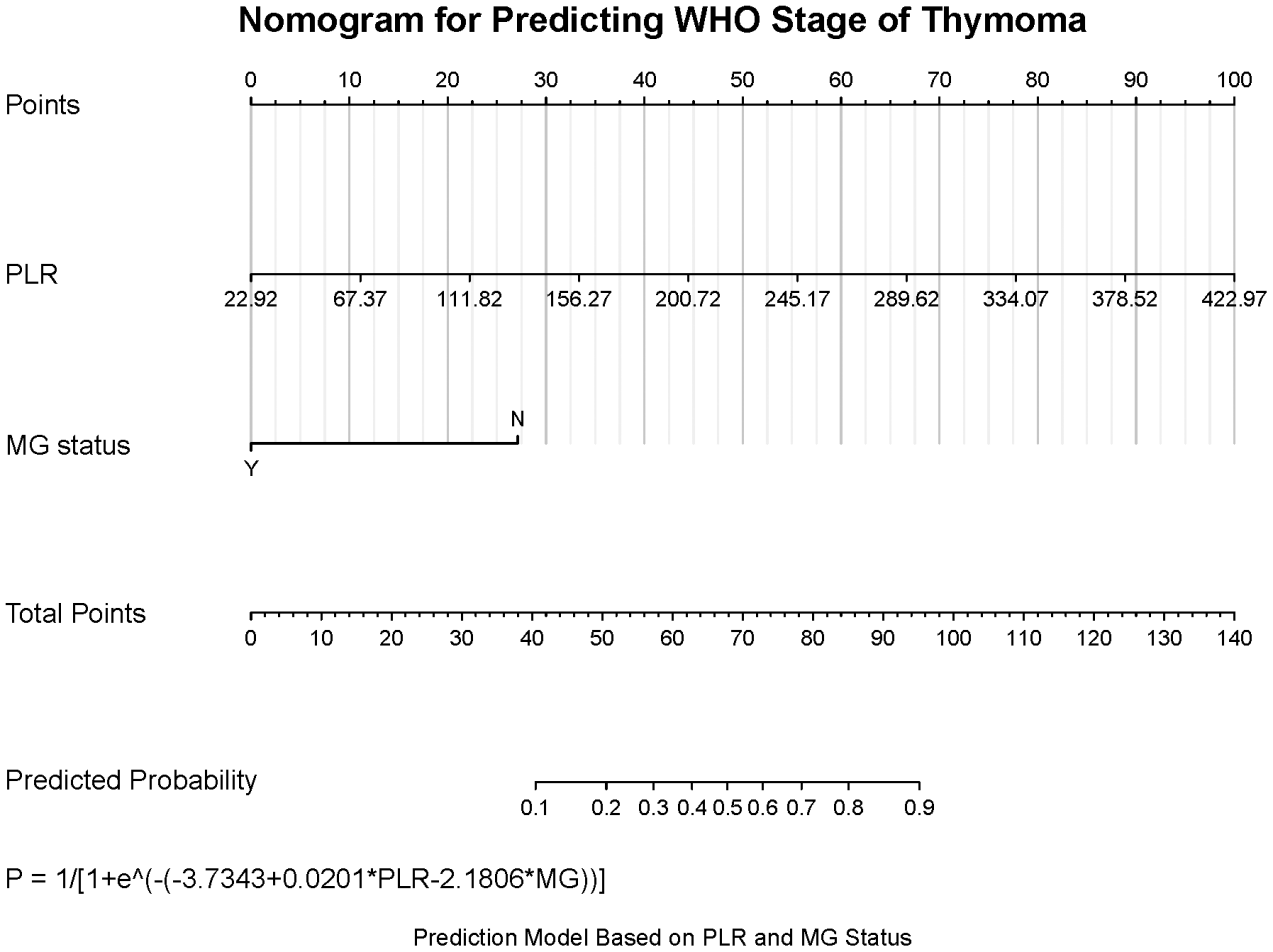
The nomogram clinical diagnostic models. The probability of thymoma prediction is calculated as: P = 1/[1+eˆ(−(−3. 7343+0.0201×PLR−2.1806×MG))], where the variableMGis defined as 1 if the patient presents with myasthenia gravis symptoms (MG = Y), and as 0 if the patient does not have myasthenia gravis symptoms (MG = N).

**Figure 5:**
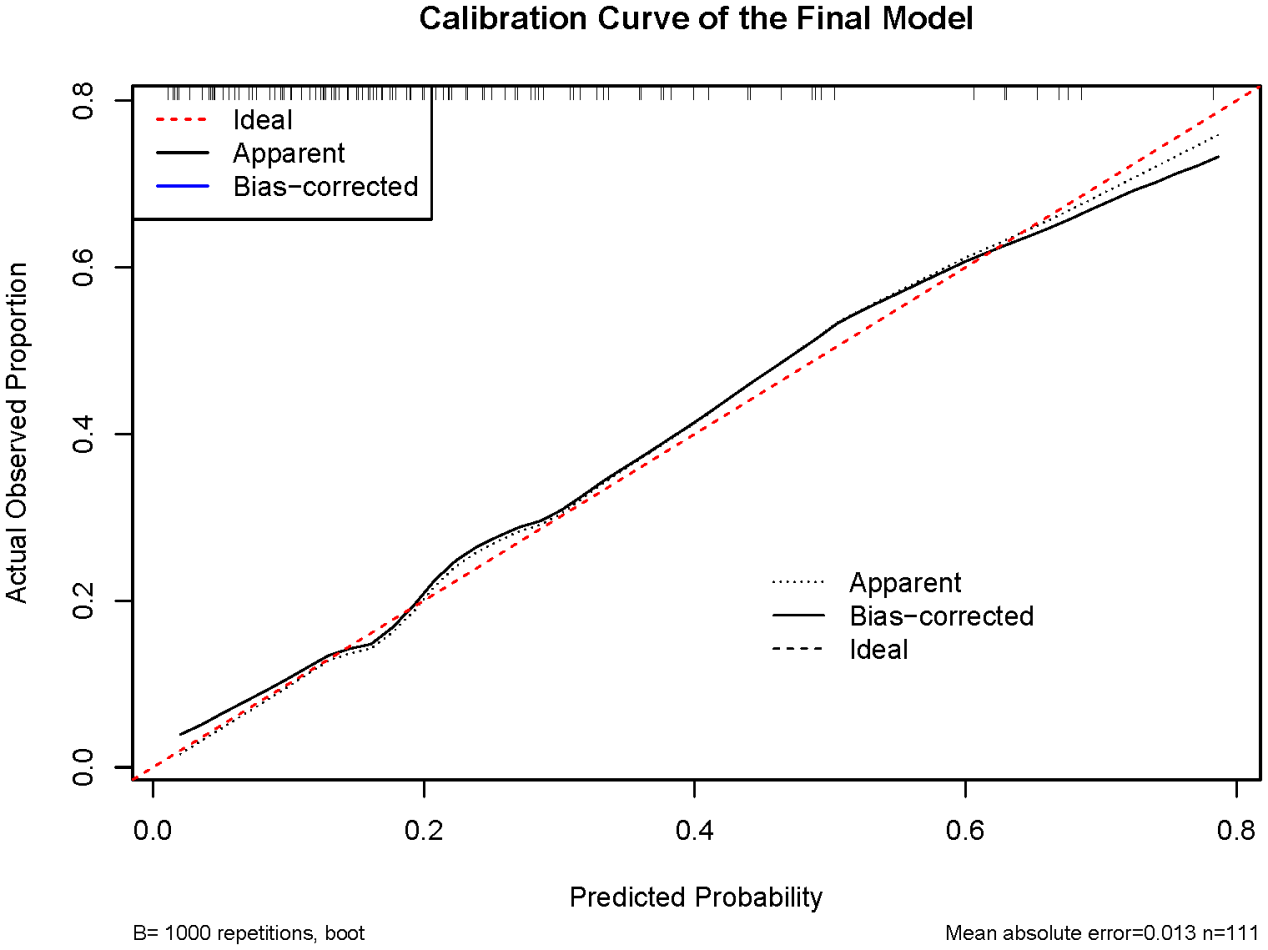
Calibration curve analysis was used for model validation. The calibration curve demonstrated good agreement between predicted probabilities and observed outcomes.

To further identify reliable diagnostic predictors, we performed stepwise regression analysis incorporating SII, NLR, PLR, age, sex, and MG status. The model combining PLR and MG status achieved the lowest AIC value (104.43). Receiver operating characteristic (ROC) curve analysis further demonstrated that the PLR + MG model exhibited favorable diagnostic performance, with an area under the curve (AUC) of 0.804 (Fig. 3). Within this model, higher PLR and the absence of MG were significantly associated with more advanced WHO staging. Accordingly, a nomogram based on PLR and MG status was developed to estimate the probability of TC, yielding the probability formula: *P* = 1 / [1 + eˆ (−(−3.7343 + 0.0201 ×PLR −2.1806 ×MG))] (Fig. 4). The Hosmer–Lemeshow goodness-of-fit test indicated good calibration of the model (*χ*^2^ = 5.76, P = 0.67) (Fig. 5). Furthermore, the model demonstrated good discriminative ability with an optimism-corrected C-index of 0.79, as determined by bootstrap validation with 1,000 resamples.

**Table 4:** Distribution profiles of SII, NLR, and PLR in WHO histological classifications. Dunn’s test was performed to further investigate specific changes in SII, NLR, and PLR among different WHO stages.

Comparison	SII			NLR			PLR		
	*Z*	*P*	P.adj	*Z*	*P*	P.adj	*Z*	*P*	P.adj
A-AB	0.42	0.68	1	−0.43	0.67	1	1.40	0.16	1
A-B1	2.37	0.02	0.27	1.66	0.10	1	2.87	0.00	0.06
AB-B1	2.67	0.01	0.11	2.63	0.01	0.13	2.30	0.02	0.32
A-B2	0.47	0.64	1	0.19	0.85	1	1.13	0.26	1
AB-B2	0.13	0.89	1	0.84	0.40	1	−0.18	0.86	1
B1-B2	−2.28	0.02	0.34	−1.74	0.08	1	−2.18	0.03	0.44
A-B3	1.28	0.20	1	0.19	0.85	1	1.93	0.05	0.80
AB-B3	1.27	0.20	1	0.77	0.44	1	1.02	0.31	1
B1-B3	−1.29	0.20	1	−1.64	0.10	1	−1.17	0.24	1
B2-B3	1.00	0.32	1	0.01	0.99	1	1.03	0.30	1
A-C	−1.23	0.22	1	−1.58	0.12	1	−0.72	0.47	1
AB-C	−2.63	0.01	0.13	−1.86	0.06	0.93	−3.34	0.00	**0**.**012**
B1-C	−4.30	0.00	**<0**.**001**	−3.76	0.00	**<0**.**001**	−4.40	0.00	**<0**.**001**
B2-C	−2.22	0.03	0.40	−2.28	0.02	0.34	−2.48	0.01	0.20
B3-C	−3.12	0.00	**0**.**027**	−2.08	0.04	0.56	−3.39	0.00	**0**.**01**

**Notes.**

NLR, neutrophil-to-lymphocyte ratio; PLR, platelet-to-lymphocyte ratio; SII, systemic immune-inflammation index; P, unadjusted *p*-value; p.adj,*p*-value adjusted for multiple comparisons using the Bonferroni method. Bold values indicate significant differences between groups (*p* < 0.05).

## Discussion

Tumor-associated inflammation plays a critical role in tumor progression. In this study, we analyzed the clinical and demographic characteristics of our cohort, including gender, age, MG status, tumor size, and WHO/Masaoka-Koga staging. MG, characterized by muscle weakness caused by autoantibodies targeting neuromuscular junction peptides (Özçıbık Işık & Turna, 2024; Perrino et al., 2024), is a classic paraneoplastic syndrome of thymoma. However, our results showed no significant associations between MG status and inflammatory indices. We further investigated the diagnostic value of preoperative SII, PLR, and NLR in distinguishing thymoma from TC among 111 completely resected TETs. Our findings revealed progressive elevations of NLR, SII, and PLR with advancing tumor stage. Increased NLR, PLR, or SII reflects elevated neutrophils/platelets or reduced lymphocytes. Systemic inflammatory responses in malignancy involve altered proportions of inflammatory cells, including enhanced neutrophil infiltration (Wang et al., 2020; Yanagiya et al., 2018). During thymoma progression, tumor-associated inflammation intensifies, promoting neutrophil recruitment through cytokine secretion (*e.g.*, interleukin family, TNF-α, VEGF) that facilitates cancer progression (Muriana et al., 2018; Okada et al., 2020). Lymphocytes play dual roles in malignancy: tumor-infiltrating lymphocytes (TILs) correlate positively with prognosis and treatment response in multiple cancers, while systemic lymphopenia reflects immunosuppressive tumor microenvironments (Chang et al., 2020; Damaj et al., 2025). The interaction between tumor cells and lymphocytes is a critical factor in immune suppression. For instance, the aberrantly expressed sHLA-G on tumor cells may directly bind to inhibitory receptors on the lymphocyte surface, delivering a potent inhibitory signal (Morandi et al., 2007).

**Table 5:** Distribution profiles of SII, NLR, and PLR among thymoma and TCs. Dunn’s test was used to explore specific changes in SII, NLR, and PLR across different risk stratifications. The results indicated significant differences in all three inflammatory factors in TC compared to both HRT and LRT.

Comparison	SII			NLR			PLR		
	*Z*	*P*	P.adj	*Z*	*P*	P.adj	*Z*	*P*	P.adj
HRT - LRT	−0.45	0.65	1.00	−0.62	0.54	1.00	−0.44	0.66	1.00
HRT - TC	−3.37	0.00	**<0.001**	−3.04	0.00	**0.01**	−3.81	0.00	**<0.001**
LRT - TC	−3.35	0.00	**<0.01**	−2.82	0.00	**0.01**	−3.86	0.00	**<0.001**

**Notes.**

NLR, neutrophil-to-lymphocyte ratio; PLR, platelet-to-lymphocyte ratio; SII, systemic immune-inflammation index; *P*, unadjusted *p*-value; p.adj, *p*-value adjusted for multiple comparisons using the Bonferroni method. Bold values indicate significant differences between groups (*p* < 0.05).

**Table 6:** Distribution profiles of SII, NLR, and PLR among thymoma and TCs. The analytical results show that SII, NLR, and PLR all exhibit significant differences between thymoma and thymic carcinoma (TC). Bold values indicate significant differences between groups (*p* < 0.05).

Overall (*N* = 111)	SII		NLR		PLR	
	Median (IQR)	*P*	Median (IQR)	*P*	Median (IQR)	*P*
Thymic carcinoma (*N* = 28)	654.07 (444.33, 796.02)	**0.006**	2.47 (1.97, 2.71)	**0.025**	172.82 (135.54, 219.98)	**<0.001**
Thymoma (*N* = 83)	393.78 (284.04, 584.42)		1.89 (1.43, 2.40)		111.97 (90.59, 149.17)	

**Notes.**

NLR, neutrophil-to-lymphocyte ratio; PLR, platelet-to-lymphocyte ratio; SII, systemic immune-inflammation index. Bold values indicate significant differences between groups (*p* < 0.05).

The higher level of SII, NLR, and PLR values observed in TCs (larger tumor size, advanced Masaoka-Koga and WHO stages) predict poorer prognosis, consistent with previous studies (Chen et al., 2017; Eraslan et al., 2021; Huang et al., 2019; Jiang et al., 2021; Jing et al., 2024; Liu et al., 2024; Nakamoto et al., 2023). Furthermore, elevated SII, NLR, and PLR have been associated with poorer prognosis in numerous other malignancies (Chen et al., 2017; Eraslan et al., 2021; Huang et al., 2019; Jiang et al., 2021; Jing et al., 2024; Liu et al., 2024; Nakamoto et al., 2023). However, there is no definitive consensus regarding which specific index exhibits superior diagnostic efficacy. Elevated SII, NLR, and PLR reflect increased neutrophils/platelets and/or decreased lymphocytes. Tumor-associated inflammation acts as a double-edged sword, but the balance between pro-tumor growth and anti-tumor immunity progressively shifts toward tumor promotion during disease progression (Greten & Grivennikov, 2019). Pro-tumor inflammation arises from interactions between tumor and immune cells within the tumor microenvironment, leading to suppressed anti-tumor lymphocyte function, reduced lymphocyte counts, and enhanced neutrophil/platelet recruitment (Petrie, Klassen & Kay, 1985; Templeton et al., 2014), which directly elevates SII, NLR, and PLR values.

The WHO classification and Masaoka-Koga staging system remain the most widely used frameworks for thymoma. Specifically, the WHO classification categorizes TETs based on the malignant differentiation of epithelial cells and lymphocyte proportion, whereas the Masaoka-Koga system stages tumors according to their invasiveness (Lee et al., 2016). Our results demonstrated more significant correlations between PLR and WHO classification, which indirectly validates the reliability of our findings. Additionally, previous studies have shown a close association between MG and thymic diseases, with MG occurring in approximately 15%–60% of thymoma patients (Wang et al., 2024; Zhang, Yu & Ke, 2022). However, multiple studies also indicate that MG is predominantly observed in patients with thymoma and is rarely present in those with TC (Kim, Koh & Minn, 2015; Kurihara et al., 2016; Li et al., 2016; Shimamura et al., 2025; Wang et al., 2023), which is consistent with the findings of our study. We developed an auxiliary diagnostic model combining PLR and MG, which demonstrates higher diagnostic efficacy compared to single-factor diagnostic models. In our model, patients without MG and with higher PLR values receive higher scores and are more likely to be diagnosed with TC rather than thymoma. In such cases, total thymectomy should be prioritized during surgery, with meticulous evaluation of tumor margins and adjacent tissue/organ involvement to achieve R0 resection, thereby improving recurrence-free survival.

This study’s retrospective design and single-center experience inherently constrain the generalizability of our conclusions, compounded by the limited cohort size (*n* = 111). As in the tumor size grouping, only 11 cases (9.9%) have tumors ≥3 cm. The small sample size may lead to unreliable results in the correlation analysis between tumor size and inflammatory indicators. Larger multi-institutional cohorts may yield more robust findings. Furthermore, our diagnostic model underwent only internal validation without external validation, which limits its generalizability. Nevertheless, the model demonstrates translational potential due to its reliance on routine hematological parameters (SII, NLR, PLR), rendering it cost-effective and readily implementable in clinical practice. The model’s simplicity facilitates multicenter validation and refinement across diverse populations.

## Supplemental Information

10.7717/peerj.21232/supp-1 Supplemental Information 1 Raw clinical data

10.7717/peerj.21232/supp-2 Supplemental Information 2 The WHOMscore scoring system

10.7717/peerj.21232/supp-3 Supplemental Information 3 R code of this analysis

## Additional Information and Declarations

### Data Availability

The following information was supplied regarding data availability: The raw data are available in the Supplemental File.
