# Supplementary material for: Correlations between inflammatory biomarkers and clinicopathological features in surgically resected thymic epithelial tumors: implications for preoperative diagnosis
Source: PeerJ. 2026 May 11;14:e21232. doi: 10.7717/peerj.21232 (PMC13175061; doi:10.7717/peerj.21232)
Supplement: Supplemental Information 2 [file peerj-14-21232-s002.docx]

| WHOMScore | | WHO classification | | | | | |
| --- | --- | --- | --- | --- | --- | --- | --- |
| Masaoka stage |  | A | AB | B1 | B2 | B3 | CA |
|  | I | 1 | 1 | 1 | 2 | 2 | 2 |
|  | II | 1 | 1 | 1 | 2 | 2 | 3 |
|  | III | 2 | 2 | 2 | 3 | 3 | 3 |
|  | IV | NA | NA | NA | 4 | 4 | 4 |
